# Does trade policy uncertainty hurt renewable energy-related sustainable development goals in China?

**DOI:** 10.1016/j.heliyon.2024.e35215

**Published:** 2024-07-25

**Authors:** Qiang Zuo, Muhammad Tariq Majeed

**Affiliations:** a Shanxi Institute for Finance Research, Taiyuan, 030000, China; b Zhong He Sheng Capital Management Co., Ltd., Taiyuan, 030000, China; c Quaid-i-Azam University, Islamabad, Pakistan

**Keywords:** Trade policy uncertainty, Renewable energy consumption, Sustainable development, China

## Abstract

Trade policy uncertainty might hamper trade flow, including the trade of green and renewable energy technologies. Therefore, this study aims to examine the asymmetric effects of trade policy uncertainty (TPU) on renewable energy consumption (REC) in China. To calculate the short- and long-term relationships between REC, TPU, national income, carbon footprints, and financial development, we used the nonlinear QARDL technique. The estimates reveal that an upsurge in TPU hurts REC in the short and long run. Conversely, a stable trade policy or a reduction in TPU increases REC in the long run. In the short run, a fall in TPU exerts no influence on REC. The findings further imply that various factors, including GDP, CO2 emissions, and financial development, contribute to long-term improvements in REC in China, both in the short and long run.

## Highlights

• The study examines the impact of TPU on REC in China.
• We utilized the nonlinear QARDL method to analyze relationships.
• Findings indicate that increased TPU negatively affects REC in the short and long run.
• Conversely, a reduction in TPU increases REC only in the long run.

## 1 Introduction

The endorsement of renewable energy to attain sustainable development objectives is strongly upheld by major global agreements such as the Kyoto Protocol of 1997, the 2015 Paris Conference, and the UN's Sustainable Agenda for 2030 [1]. These pacts demand that countries exert significant resources in their energy policy to reduce carbon emissions. The European Union (EU), which committed to reducing pollution by 8 % from 2008 to 2012, has achieved its objective. To accomplish this, the European Parliament introduced several initiatives emphasizing renewables, aiming for renewable resources to account for 22 % more electricity generation and 12 % more primary energy demand by 2010 [2]. Energy-saving technology and the renewable energy sector development have been used to increase jobs and energy independence while advancing ecological aims [3].

The discussion about climate change and its effects, following the 2007 release of the 4th IPCC assessment report, has elevated the priority of renewable energy. If other nations take up the mantle, the EU Commission will establish the objective of a 30 % reduction in carbon production [4]. The EU Commission urges Europe to take the initiative in reducing its carbon footprint. Broadly speaking, the benefits of using renewables in electricity generation, heat, and logistics within the framework of environmental benefits, resource protection, and import autonomy presently seem to be beyond dispute. However, the total financial costs of supporting green energy in these areas continue to be an issue [5].

Entrepreneurs, trade advisers, legislators, and other concerned parties are becoming more and more interested in trade talks and ideas for a fresh perspective on trade policy. The future of global trade is unclear as a result of such attempts [6]. Effectively, it has been determined that trade policy uncertainty (TPU) is a significant reason for economic policy unpredictability. After the vote for Brexit and Trump's victory, the world's important economies, i.e., the USA and the UK, have decreased their preference for low protectionist measures and strong trade treaties. Such ambiguity spread throughout established and emerging economies while creating new international worries due to the inevitable bargaining and departure from long-standing trade accords and the 2018 trade tensions [7].

Trade policy significantly impacts domestic and international investments by influencing the marketplace size for a firm's products [8,9]. Trade policy impact on businesses has expanded over time. Policymakers worldwide are paying attention to the expanding TPU and its related implications. As per the World Bank's research, “confidence and investment might be dramatically damaged by a rapid spike in policy uncertainty, driven, for example, by considerable new trade barriers between major countries". For instance, a three-year period of retaliatory tariff policies between the USA and China resulted in a significant decline in worldwide revenue. The possible detrimental impacts of TPU on business and economic development are supported by Handley & Limao [10].

TPU impacts enterprises' immediate actions, including price and inventory management. TPU impacts long-term investment choices, such as market entrance [11]. Handley & Limo [10] support the moderating trade impact of TPU. Economic and ecological ramifications follow from such a result. Environmental deterioration will increase if trade declines in industries that promote environmentally friendly and green technology, and vice versa [12,13]. To determine the total impacts of trade, it is useful to include possible disruptions to the TPU. According to the available studies, TPU has wider consequences in an open market economy. For instance, China's entry into the WTO, Brexit, and the 2018 trade tensions all affected TPU, which in turn might influence profitability, demand, production, and a variety of other macroeconomic outcomes [14]. Economic outcomes, rising prices, and financing have been the key topics of the empirical research that has already been conducted [15,16].

TPU might theoretically have a variety of negative environmental impacts. If the trade-in green and ecologically favorable technology is stifled, growing TPU might hurt environmental sustainability [17]. In contrast, TPU may benefit the environment if it hinders the exchange of traditional forms of energy and technology [18]. Therefore, determining the true effect of TPU on the ecosystem and renewable energy investment becomes an empirical question. Nevertheless, examining the relationship between TPU and ecological quality offers a partial view of sustainable growth objectives. Notably when the rate of growth and pollutants are combined. Cutting pollution in this circumstance will also jeopardize expectations for economic development [19]. Decoupling pollution and growth is still essential. Increasing renewable energy consumption (REC) and production has become crucial in the contemporary age to guarantee sustainable economic development [20].

In the empirical literature on REC, there is a consensus that trade liberalization has a crucial role in determining REC [21,22]. On the other hand, the previous research emphasized the significance of TPU for socio-economic aspects [23], energy dynamics [9], and environmental issues [24]. Despite the significance of TPU in shaping energy dynamics, empirical studies are unavailable concerning the nexus between TPU and REC. Moreover, the outcomes of the past studies are inconclusive due to the reliance on traditional estimation approaches. Thus, we have identified the following gaps in the literature. First, no past study has investigated the impact of TPU on REC. Second, no past study has estimated the nexus between China's TPU and REC. Third, ignoring asymmetric analysis is another weakness in the past literature. Lastly, in many past studies, contemporary literature has relied on conventional and linear estimation approaches such as ARDL, contributing to inconclusive and insignificant estimates.

The primary motive of this study is to estimate the role of TPU in impacting REC. While achieving this motive, the study makes the following contribution to the current body of knowledge. First, as far as we know, this analysis is the inaugural effort to examine the impact of TPU on REC in China. Thus, this study makes a significant contribution to the existing literature by furnishing both empirical evidence and theoretical underpinnings, thereby laying a foundation for future research in this field. Second, China is the largest investor in the renewable energy sector and was recently involved in a trade war with the USA, which has induced China to change its trade policy. Hence, examining the nexus in the context of China can prove vital in understanding the complex dynamics between TPU and REC across the globe, particularly in the developing world. Third, estimating the nexus by depending on the asymmetric assumption is another value addition to the literature as the macro variables, particularly those linked to business cycles, follow the nonlinear path across time. Since trade policy is vulnerable to external shocks, it is justifiable that the nonlinear impact of TPU on REC will be seen. Fourth, employing QARDL to estimate the above-stated nexus can help us identify the impact of TPU on different levels of REC in the short and long run. Lastly, the results significantly suggest important policy guidelines to the concerned stakeholders.

### 1.1 Literature review

Renewable energy sources are widely acknowledged as a driver of superior environmental quality and sustainable development. Thus, the literature on the factors of renewable energy is growing. As per the given works, REC is mostly determined by economic growth, technological progress, and financial development [25]. While some other studies have also highlighted the significance of trade openness in promoting REC [[21], [22], [23], [24], [25], [26]]. Following the initial effort by Baker et al. [27] that laid the foundation for future works, several researchers have recognized the significance of economic policy uncertainty (EPU), including monetary, tax, and trade policies. Empirics are interested in understanding the role of diverse policy uncertainties in shaping the energy structure [28,29].

In the context of renewable energy, most economists have considered EPU as a potential determinant of REC but have overlooked the importance of TPU. For instance, a growing number of studies have confirmed that EPU significantly influences renewable energy [30]. Particularly, it is observed that EPU hinders investment in renewable energy projects [31], which significantly hurts renewable energy use. EPU has a vital role in discouraging enterprises from tolerating more risks that can reduce the prospect of future earnings. As a result, the demand for renewable energy can be reduced. Ivanovski and Marinucci [32] observed an inverse connection between EPU and renewable energy in the long run. Zeng and Yue [33] highlighted that EPU, and renewable energy use are inversely related to each other in the context of BRICS economies. According to Sohail et al. [34], monetary policy uncertainty is crucial in curtailing short- and long-term REC. In contrast, some researchers observed a positive role of EPU in fostering renewable energy investment. For instance, Liu et al. [35] confirmed that investing more in renewable energy projects became possible due to a rise in EPU. As per the work of Shang et al. [36], any uncertainty in climate policy in the USA is the factor behind rising investment in the renewable energy sector.

A growing body of literature has estimated the nexus between trade openness/globalization and REC [[21], [22], [23], [24], [25], [26]]. However, as already stated, the nexus between TPU and renewable energy has not received much attention. Due to the increased popularity of trade protectionism, empirics have started to focus on the role of external risks, e.g., TPU, in shaping the energy dynamics of different nations and economies. Particularly, when uncertainty is prevalent, it is crucial to consider the impact of trade policy and the uncertainties attached to such a policy. More precisely, there are researchers who shed light on the dynamics of TPU and the energy markets nexus [37,38]. By collecting data from Chinese industrial enterprises, Yang and Hong [39] highlighted that TPU helps reduce the energy intensity in these enterprises. Likewise, Wang and Wu [23] revealed that TPU in China is detrimental to energy consumption in China. Xie et al. [38], in the context of three sectors of the USA, examined the nexus between TPU and energy consumption. Their findings highlight that TPU is vital in determining energy consumption in residential and commercial sectors. Li et al. [9] revealed that energy efficiency improved in China due to a decline in TPU. Some have examined trade policy's impact on energy demand [38].

From the above discussions, it is derived that trade policy uncertainties can play a vital role in the development of the renewable energy sector and, ultimately, its consumption. The contemporary literature regarding trade policy uncertainties has mainly emphasized the impact of these uncertainties on energy structures or markets. However, the literature on the effect of TPU on REC is in its initial stages. Thus, to fill this gap, we need to investigate TPU and REC nexus.

### 1.2 Theoretical framework and hypothesis development

Contemporary literature on international trade agrees that nations can enjoy several static welfare benefits by being involved in trade. An increase in flow utility signifies these benefits because of the countries' involvement in global markets. The diverse opportunity costs regarding manufacturing goods provide the foundation of comparative and welfare advantages [40,41]. New trade models, which rely on the work of Helpman and Krugman [42], show strong faith in product diversification and rising returns to scale, leading to welfare benefits from trade. Lastly, as per the works of Melitz [43] and Bernard et al. [44], which propose the models of heterogeneous firms in different product marketplaces, the primary reason behind intra-industry resource distributions is that the most proficient companies are not selected randomly before entering global trade markets. Companies or enterprises that work efficiently successfully enter the global market, while those that work less efficiently are forced to shut down because of increased competition. As a result, an increased average industrial output maximizes welfare benefits.

As stated above, comparative advantage is the primary reason behind international trade. Thus, international trade can play a vital role in enhancing REC due to the following reasons. First, the minerals used in the development of renewable energy innovations, equipment, and projects are located in different parts of the world, international trade is the only option through which these minerals can be reached in every corner of the world. This would result in the increased production of renewable energy and ultimately boost its consumption [45]. Second, renewable energy technologies are crucial in fostering REC. However, these technologies can be spread across the globe with the help of international trade, allowing developing nations to access state-of-the-art equipment and expertise that are not indigenously available. This spread of technologies helps lower production costs, encourages innovation and economies of scale, and ultimately brings down the overall cost of renewable energy generation and boosts its consumption [46]. Third, based on the interdependence hypothesis, international trade can open the door for international collaborations in the field of renewable energy that can provide opportunities for nations to adopt renewable energy policies and standards [47]. This can create a more feasible environment for the investment in renewable energy. Fourth, to overcome the resource constraints, international trade can play a vital role and allow the nations to specialize in generating renewable energy tools and equipment where they have comparative advantages, supporting the well-organized global supply chains and encouraging the growth of the renewable energy sector [48].

**Fig. 1:**
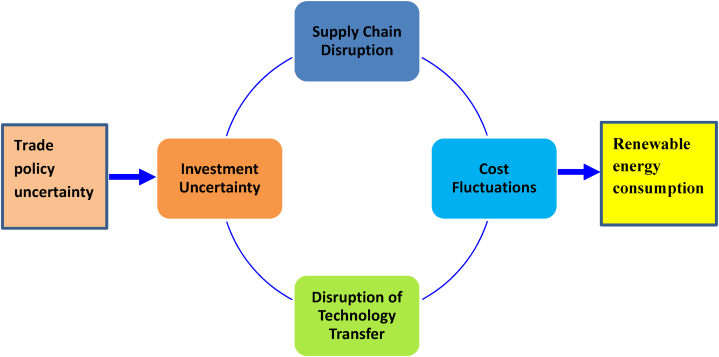
Conceptual framework.

Since trade is crucial for fostering renewable energy production and consumption, any uncertainty in trade policy can hinder REC (see, Fig. 1). According to “real options approach” due to uncertainty an enterprise can postpone its investment and innovation decisions because it requires more information before deciding about such matters [49]. Moreover, uncertainty can increase the risks attached to international trade. As a result, risk-averting traders trade less to avoid any future loss. Considering these arguments, we can state that TPU can lower the trade of critical minerals, hinder the spread of renewable energy technologies, and decrease the chances of international collaboration in the renewable energy sector. Thus, we have developed the following hypothesis.

*Hypothesis: Trade policy uncertainty can hinder renewable energy consumption by hurting the trade that promotes renewable energy development.*

### 1.3 Model and methods

We have utilized the QARDL method proposed by Cho et al. [50] to explore the asymmetries between concerning variables. This technique is ideal over linear models for various reasons. Firstly, it considers locational asymmetries in which outcomes can only rely on the dependent variables. The ARDL fails to capture the asymmetric associations between variables, so QARDL is considered a more suitable approach. In addition, the QARDL method can examine short-term and long-term dynamics across various quantile ranges. Further, this technique can also control the endogeneity issue as it includes the lagged value of the dependent variable on the right-hand side of the equation. This method is also effective in detecting structural breaks. Numerous scholars have employed this method to investigate diverse economic phenomena [51,52]. Therefore, as it is an advanced technique, we have used the QARDL to explore the connections between REC and TPU, GDP, CO2, and financial development. The simplest form of the QARDL model is expressed as follows:

(1) $${\text{REC}}_{\;t}=\mu +\sum_{i=1}^{n1}\sigma_{\;{\text{REC}}_{i}}{\text{REC}}_{t-i}+\sum_{i=0}^{n2}\sigma_{\;{\text{TPU}}_{i}}{\text{TPU}}_{t-i}+\sum_{i=0}^{n3}\sigma_{\;{\text{GDP}}_{i}}{\text{GDP}}_{t-i}+\sum_{i=0}^{n4}\sigma_{\;{\text{CO}2}_{i}}{\text{CO}2}_{t-i}+\sum_{i=0}^{n5}\sigma_{\;{\text{FD}}_{i}}{\text{FD}}_{t-i}+\varepsilon_{t}$$

where $\varepsilon_{t}$ is identified as REC_t_ -E [RECt/Ft − 1] with Ft – 1 is the lowest σ–field determined by (TPU_t_, GDP_t_, CO2_t_, FD_t_, TPU_t-1_, GDP_t-1_, CO2_t-1_, FD_t-1_}, and n1 ….n5 denotes the orders of lag. Eq. (1) classifies that trade policy uncertainty, GDP per capita, CO2 emissions, and financial development are denoted by TPU_t_, GDP_t_, CO2_t_, FD_t_, while REC_t_ represents renewable energy consumption. To transform the basic equation (1) into the QARDL format, we adopt the methodology introduced by Cho et al. (2015).

(2) $$Q_{\;{\text{REC}}_{t}}=\mu \left(\tau \right)+\sum_{i=1}^{n1}\sigma_{\;{\text{REC}}_{i}}\left(\tau \right){\text{REC}}_{t-i}+\sum_{i=0}^{n2}\sigma_{\;{\text{TPU}}_{i}}\left(\tau \right){\text{TPU}}_{t-i}+\sum_{i=0}^{n3}\sigma_{\;{\text{GDP}}_{i}}\left(\tau \right){\text{GDP}}_{t-i}+\sum_{i=0}^{n4}\sigma_{\;{\text{CO}2}_{i}}\left(\tau \right){\text{CO}2}_{t-i}+\sum_{i=0}^{n5}\sigma_{\;{\text{FD}}_{i}}\left(\tau \right){\text{FD}}_{t-i}+\varepsilon_{t}\left(\tau \right)$$

In Eq. (2), the value of τ lies between 0 and 1 and indicates the quantile level. Additionally, there is a chance of serial correlation within Eq. (2), and a nonlinear QARDL may be employed. However, for our analysis, we concentrate only on the assumption of nonlinearity. As such, we utilized the partial sum procedure to distinguish the TPU variable into its negative and positive components. Following Shin et al. [53], we have split the only TPU for nonlinear analysis.

(3a) $${\text{TPU}}_{t}^{+}=\sum_{n=1}^{t}{\Delta \text{TPU}}_{t}^{+}=\sum_{n=1}^{t}\max\;\left({\Delta \text{TPU}}_{t}^{+},0\right)$$

(3b) $${\text{TPU}}_{t}^{-}=\sum_{n=1}^{t}{\Delta \text{TPU}}_{t}^{-}=\sum_{n=1}^{t}\min\;\left({\Delta \text{TPU}}_{t}^{\;-},0\right)$$

Afterwards, we return to Eq. (2) and substitute the upward and downward variations of TPU to obtain Eqs. (3a), (3b):

(4) $$Q_{\;{\Delta \text{REC}}_{t}}=\mu +\rho {\text{REC}}_{t-1}+\beta_{\text{TPU}}^{+}{\text{TPU}}_{t-1}^{+}+\beta_{\text{TPU}}^{-}{\text{TPU}}_{t-1}^{-}+\beta_{\text{GDP}}{\text{GDP}}_{t-1}+\beta_{\text{CO}2}{\text{CO}2}_{t-1}+\beta_{\text{FD}}{\text{FD}}_{t-1}+\sum_{i=1}^{n1}\pi_{\;{\text{REC}}_{i}}\Delta {\text{REC}}_{t-i}+\sum_{k=0}^{n2}{\pi^{+}}_{\;{\text{TPU}}_{i}}{\Delta \text{TPU}}_{t-i}^{+}+\sum_{k=0}^{n3}{\pi^{-\;\text{on}\;\text{GE}\;\text{een}\;\text{ods}.\;\text{Contrariwise},}}_{\;{\text{TPU}}_{i}}{\Delta \text{TPU}}_{t-i}^{-}+\sum_{i=0}^{n4}\pi_{\;{\text{GDP}}_{i}}{\Delta \text{GDP}}_{t-i}+\sum_{i=0}^{n5}\pi_{\;{\text{CO}2}_{i}}{\Delta \text{CO}2}_{t-i}+\sum_{i=0}^{n4}\pi_{\;{\text{FD}}_{i}}{\Delta \text{FD}}_{t-i}+\varepsilon_{t}\left(\tau \right)$$

Representing Eq. (2) using the QARDL-ECM format within the nonlinear QARDL framework has the potential to alleviate correlations through the projection of εt onto concerned variables. Therefore, Eq. (4) shows the nonlinear QARDL-ECM version as:

(5) $$Q_{\;{\Delta \text{REC}}_{t}}=\mu \left(\tau \right)+\rho \left(\tau \right)\left({\text{REC}}_{t-1}-\beta_{\text{TPU}}^{+}\left(\tau \right){\text{TPU}}_{t-1}^{+}-\;\beta_{\text{TPU}}^{-}\left(\tau \right){\text{TPU}}_{t-1}^{-}-\beta_{\text{GDP}}\left(\tau \right){\text{GDP}}_{t-1}-\beta_{\text{CO}2}\left(\tau \right){\text{CO}2}_{t-1}-\beta_{\text{FD}}\left(\tau \right){\text{FD}}_{t-1}\right)+\sum_{i=1}^{n1}\pi_{\;{\text{REC}}_{i}}\left(\tau \right)\Delta {\text{REC}}_{t-i}+\sum_{i=0}^{n2}{\pi^{+\text{on}\;\text{GE}\;\text{een}\;\text{ods}.\;\text{Contrariwise},}}_{\;{\text{TPU}}_{i}}\left(\tau \right){\Delta \text{TPU}}_{t-i}^{+}+\sum_{i=0}^{n3}{\pi^{-\;\text{on}\;\text{GE}\;\text{een}\;\text{ods}.\;\text{Contrariwise},}}_{\;{\text{TPU}}_{i}}\left(\tau \right){\Delta \text{TPU}}_{t-i}^{-}+\sum_{i=0}^{n4}\pi_{\;{\text{GDP}}_{i}}\left(\tau \right){\Delta \text{GDP}}_{t-i}+\sum_{i=0}^{n5}\pi_{\;{\text{CO}2}_{i}}\left(\tau \right){\Delta \text{CO}2}_{t-i}+\sum_{i=0}^{n6}\pi_{\;{\text{FD}}_{i}}\left(\tau \right){\Delta \text{FD}}_{t-i}+\varepsilon_{t}\left(\tau \right)$$

The short-term effect of ${\text{TPU}}^{+}$_t_, ${\text{TPU}}^{-}$_t_, ${\text{GDP}}_{t}$, ${\text{CO}2}_{t}$, and ${\text{FD}}_{t}$ are represented by $\pi^{*}\sum_{j=1}^{n2}\;\pi_{j}$, $\pi^{*}\sum_{j=1}^{n3}\;\pi_{j}$, $\pi^{*}\sum_{j=1}^{n4}\;\pi_{j}$, $\pi^{*}\sum_{j=1}^{n5}\;\pi_{j}$, $\pi^{*}\sum_{j=1}^{n6}\;\pi_{j}$, respectively. The long-run estimates for trade policy uncertainty, GDP, CO2 emissions, and financial development are explained through $\beta_{\text{TPU}}^{-}*=-\frac{\beta^{-}\text{TPU}}{p},\beta_{\text{TPU}}^{+}*=-\frac{\beta^{+}\text{TPU}}{p},\beta_{\text{GDP}}*=-\frac{\beta \text{GDP}}{p}$, $\beta_{\text{CO}2}*=-\frac{\beta \text{CO}2}{p}$, and $\beta_{\text{FD}}*=-\frac{\beta \text{FD}}{p}$, correspondingly. In order to confirm the long and short-run nonlinear effects of TPU on REC, the estimation of ρ, which is linked to the REC parameter in Eq. (5), and yields a significantly negative result. Additionally, the Wald test can confirm the existence of long-run (short-run) asymmetric effects if it rejects the null hypothesis of $\beta_{\text{TPU}}^{+}$ = $\beta_{\text{TPU}}^{-}$ ($\pi_{\text{TPU}}^{+}$ = $\pi_{\text{TPU}}^{-}$).

### 1.4 Justification for QARDL

The QARDL approach is considered superior to other linear approaches for four reasons. First, due to the derivation of the parameters by relying on the location of the outcome variable, REC, within the “conditional distribution” this technique allows for the “location-based asymmetry” [54]. Second, this approach helps identify the short and long-run links between TPU and REC for several quantiles [55]. Third, as per the available literature, linear and traditional ARDL framework and Johansen cointegration approach have confirmed no cointegration between specific variables [56]. These insignificant outcomes may be due to the wrong selection of econometric techniques despite the presence of “quantile-varying cointegration coefficients” for the short run, suggesting that variables move simultaneously in the long run [57]. Additionally, as a result of shocks, the cointegration coefficient may differ among creative quantiles as per the QARDL approach. Lastly, in comparison to other nonlinear techniques, e.g., the “NARDL”, which relies on the assumption that nonlinearity is defined exogenously wherein the threshold is set at zero rather than chosen through a data-driven process.

### 1.5 Data and descriptive analysis

**Fig. 2:**
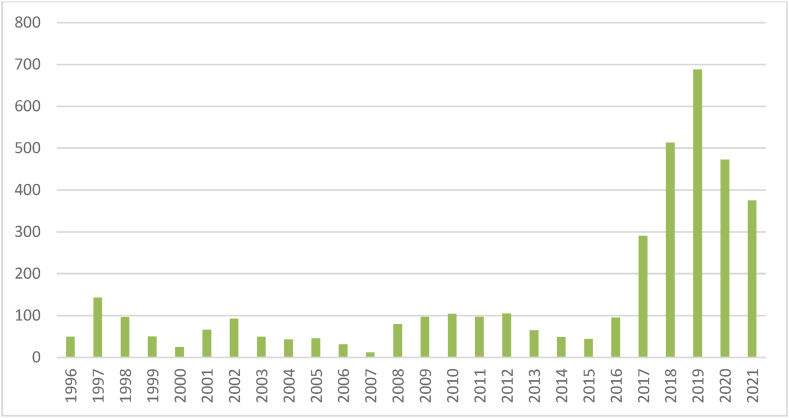
Trend of trade policy uncertainty.

**Fig. 3:**
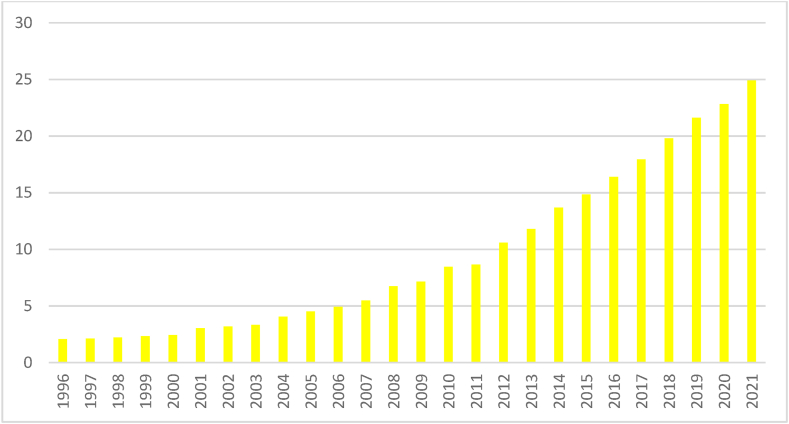
Trend of REC

Our study has collected annual data series from 1996 to 2020 to inspect the nexus between TPU and REC. Table 1 provides information on the symbols used for variables, definitions, and descriptive statistics. REC is a dependent variable, which is measured as the energy consumption from all sources, including renewables and nuclear energy. An index is used for TPU. The data for REC is attained from the EIA and TPU is collected from Baker, Bloom, and Davis. Following existing literature, this study incorporated additional control variables, namely GDP per capita, financial development, and CO2 emissions, in line with prior research [58,59]. Chao et al. [58] documented a positive correlation between GDP per capita and REC. They also argue that economies with high GDP per capita prefer investing more in renewable energy technologies due to their high financial capability, which increases REC. GDP per capita is measured at constant 2015 US$. Financial development is another pivotal control variable. Zhang et al. [60] illustrated that financial development enhances the capacity to invest in renewable energy projects that can positively augment REC. Moreover, easy access to financial markets and innovative financing mechanisms can reduce the financial cost of renewable energy projects, further boosting REC. Various proxy measures have been employed in the current literature to gauge financial development. Our study follows Alsagr [61], who measures financial development at the aggregate level through the financial development index. Following Xu & Ullah [54], CO2 emission is also a control variable. The study argues that economies with higher CO2 are more inspired to invest in renewable energy projects. CO2 emissions is measured in metric tons per capita. CO2 and GDP data series are sourced from the WDI, while the series for FD is gathered from the IMF. Key variable trends are shown in Fig. 2, Fig. 3. The descriptive statistics display mean values as: 1.786 for REC, 4.326 for TPU, 8.393 for GDP, 5.263 for CO2, and 0.497 for FD. The J-B test is used to detect the normality of variables. The estimates of J-B test display that TPU series is normally distributed while REC, GDP, CO2, and FD series are not distributed normally.

**Table 1:** Definitions and descriptive statistics.

Variables	Definitions	Mean	Median	Max	Min	Std. Dev.	Skewness	Kurtosis	Jarque-Bera	Prob.
REC	Total energy consumption from nuclear, renewables, and other (quad Btu)	1.786	1.843	3.078	0.584	0.839	−0.014	1.571	8.517	0.014
TPU	Trade policy uncertainty index	4.326	4.274	6.656	0.993	1.056	0.136	3.593	1.771	0.412
GDP	GDP per capita (constant 2015 US$)	8.393	8.458	9.247	7.379	0.600	−0.142	1.623	8.231	0.016
CO2	CO2 emissions (metric tons per capita)	5.263	5.428	8.512	2.508	2.004	−0.18	1.445	10.61	0.005
FD	Financial development index	0.497	0.515	0.670	0.342	0.101	0.097	1.588	8.462	0.015

## 2 Empirical results and discussion

In the first step, the unit root properties of data are checked using the ADF and ZA unit root tests. Table 2 reported the final decisions of both stationarity tests. The ADF test reports that only FD is level stationary, while ZA test reports that three series are level stationary (i.e., TPU, GDP, and FD). REC, TPU, GDP, and CO2 series are reported as I(1) in the ADF test, while REC and CO2 are reported I(1) in the ZA test. In the second step, the nonlinearities of the concerned variables are confirmed through the BDS test. The outcomes of BDS tests are reported in Table 3. BDS tests confirm that the concerned variables are nonlinear. These results support the use of an asymmetric QARDL approach for estimation. In the third step, the model was regressed using a nonlinear QARDL technique. Table 4 reports the findings of the nonlinear QARDL model.

**Table 2:** Results of unit root tests.

	ADF			ZA				
	I(0)	I(1)	Decision	I(0)	Break date	I(1)	Break date	Decision
REC	−1.785	−3.205**	I(1)	−2.875	2003 Q1	−4.587**	2019 Q3	I(1)
TPU	−1.842	−7.652***	I(1)	−4.758**	2016 Q3			I(0)
GDP	−1.654	−2.875*	I(1)	−6.879***	1999 Q2			I(0)
CO2	−0.232	−2.614*	I(1)	−1.654	2015 Q2	−4.651**	2019 Q1	I(1)
FD	−2.754*		I(0)	−4.356*	2005 Q1			I(0)

**Table 3:** Results of BDS test.

	REC			TPU		
Dimension	BDS	S.E	z-Statistic	BDS	S.E	z-Statistic
2	0.206***	0.004	50.33	0.101***	0.008	11.08
3	0.347***	0.006	53.79	0.166***	0.014	11.40
4	0.444***	0.008	58.16	0.202***	0.016	11.60
5	0.512***	0.008	64.65	0.231***	0.017	12.61
6	0.559***	0.008	73.69	0.228***	0.016	12.80

**Table 4:** Nonlinear QARDL estimates of REC.

Quantiles	ECM	Constant	Long-run estimates					Short-run estimates						
(τ)	ρ(τ)	c(τ)	$\beta_{\text{TPU}}^{+}\left(\tau \right)$	$\beta_{\text{TPU}}^{-}\left(\tau \right)$	$\beta_{\text{GDP}}\left(\tau \right)$	$\beta_{CO2}\left(\tau \right)$	$\beta_{\text{FD}}\left(\tau \right)$	$\pi_{\text{TPU}}^{+}\left(\tau \right)$	$\pi_{\text{TPU}}^{-}\left(\tau \right)$	${\pi 0}_{\text{GDP}}\left(\tau \right)$	${\pi 1}_{\text{GDP}}\left(\tau \right)$	${\pi 0}_{CO2}\left(\tau \right)$	${\pi 1}_{CO2}\left(\tau \right)$	$\pi_{\text{FD}}\left(\tau \right)$
0.05	−0.343^c^	20.76^a^	−0.057	−0.108	4.190^a^	−0.227	0.531	−0.016	−0.010	2.292^a^	2.276^a^	0.401^a^	0.412^a^	1.204
	(-1.860)	(10.47)	(-1.392)	(-1.022)	(8.894)	(-1.036)	(0.221)	(-0.644)	(-1.143)	(7.692)	(7.574)	(5.811)	(5.486)	(1.061)
0.10	−0.277^c^	18.95^a^	−0.010	−0.102	3.103^a^	−0.165	1.397	−0.012	−0.000	2.322^a^	2.575^a^	0.343^a^	0.364^a^	1.995
	(-1.890)	(4.570)	(-0.358)	(-1.357)	(4.361)	(-1.475)	(1.620)	(-1.601)	(-0.035)	(8.544)	(8.468)	(6.305)	(7.274)	(1.044)
0.20	−0.203^b^	17.76^a^	−0.024	−0.101	2.380^a^	−0.100	1.703^a^	−0.005^c^	−0.003	2.378^a^	2.053^a^	0.410^a^	0.419^a^	1.841
	(-2.082)	(5.155)	(-1.421)	(-1.554)	(5.148)	(-1.002)	(3.911)	(-1.830)	(-1.064)	(12.36)	(12.21)	(8.300)	(8.520)	(1.271)
0.30	−0.222^b^	16.57^a^	−0.019	−0.091^b^	2.225^a^	−0.049	1.359^a^	−0.007^a^	−0.002	2.390^a^	2.378^a^	0.417^a^	0.425^a^	1.796
	(-2.417)	(5.765)	(-1.355)	(-2.160)	(5.686)	(-1.495)	(3.212)	(-2.668)	(-0.968)	(10.38)	(10.30)	(7.475)	(7.718)	(1.580)
0.40	−0.230^b^	15.61^a^	−0.021^c^	−0.088^a^	2.095^a^	−0.026	1.271^a^	−0.008^b^	−0.001	2.553^a^	2.242^a^	0.414^a^	0.425^a^	1.937^b^
	(-2.392)	(5.741)	(-1.734)	(-4.761)	(5.641)	(-0.864)	(3.073)	(-2.253)	(-0.550)	(9.470)	(9.438)	(6.386)	(6.800)	(2.242)
0.50	−0.220^b^	14.20^a^	−0.027^b^	−0.074^a^	1.925^a^	−0.033	0.978^b^	−0.008^b^	−0.001	2.369^a^	2.355^a^	0.426^a^	0.435^a^	2.045^a^
	(-2.255)	(5.637)	(-2.323)	(-4.192)	(5.531)	(-1.033)	(2.405)	(-2.111)	(-0.459)	(9.335)	(9.304)	(6.284)	(6.642)	(3.876)
0.60	−0.286^c^	13.55^a^	−0.018^b^	−0.052^c^	1.854^a^	−0.051	0.830^b^	−0.011^b^	−0.001	2.430^a^	2.421^a^	0.378^a^	0.393^a^	1.516^a^
	(-1.826)	(4.383)	(-2.432)	(-1.682)	(4.382)	(-1.538)	(2.317)	(-2.355)	(-0.338)	(6.740)	(6.730)	(5.525)	(6.158)	(3.322)
0.70	−0.306^c^	10.70^b^	−0.013^b^	−0.016^c^	1.484^b^	0.045	0.658^b^	−0.010^c^	−0.001	2.472^a^	2.461^a^	0.339^a^	0.351^a^	1.329^a^
	(-1.860)	(2.430)	(-2.086)	(-1.729)	(2.517)	(1.405)	(2.054)	(-1.930)	(-0.387)	(5.010)	(5.014)	(3.281)	(3.595)	(3.052)
0.80	−0.374^c^	9.360^b^	−0.015^b^	−0.005^c^	1.308^b^	0.078^b^	0.583^b^	−0.005^c^	−0.001	1.006^b^	1.997^b^	0.174^b^	0.185^c^	1.616^a^
	(-1.763)	(2.049)	(-2.428)	(-1.845)	(2.144)	(2.223)	(1.998)	(-1.769)	(-0.134)	(1.998)	(1.992)	(2.274)	(1.850)	(2.957)
0.90	−0.396^c^	3.200^b^	−0.032^a^	−0.031^c^	0.481^b^	0.227^c^	0.357^b^	−0.013^c^	−0.002	1.024^b^	1.608^b^	0.282^a^	0.281^a^	0.878^c^
	(-1.921)	(2.101)	(-2.633)	(-1.957)	(2.249)	(1.778)	(1.991)	(-1.805)	(-0.214)	(2.302)	(2.265)	(3.265)	(3.621)	(1.914)
0.95	−0.410^b^	2.327^b^	−0.047^a^	−0.045^b^	0.104^b^	0.557^c^	0.029^b^	−0.026^c^	−0.014	1.052^b^	1.401^b^	0.105^b^	0.085^b^	0.915^c^
	(-2.225)	(2.121)	(-3.972)	(-2.100)	(2.291)	(1.669)	(2.091)	(-1.881)	(-0.718)	(2.532)	(2.219)	(2.504)	(2.422)	(1.932)

Note: T-stats in parentheses.

a p < 0.01.

b p < 0.05.

c p < 0.1.

The ECM parameters are revealed to be negative and significant at all quantiles. This finding infers that the variables tend to converge toward long-run equilibrium in China. In this model, the impact of nonlinear shocks of TPU on REC is examined in the long & short run. The findings indicate that a positive shock in TPU has a significant and negative effect on REC in China in the long run, specifically at quantiles 0.40 to 0.95. The findings align with our hypothesis. However, there is no significant association between positive shocks in TPU and REC at quantiles 0.05 to 0.30. On the other hand, a negative shock in TPU is significantly and positively associated with REC in China at quantiles 0.30 to 0.95 in the long run, indicating that it enhances REC at these quantiles. Conversely, negative shocks in TPU have no impact on REC at the lowest quantiles, i.e., 0.05 to 0.20. These results are reinforced by Wang & Wu [23], who indicate that TPU reduces energy consumption in China.

The study's most important results confirm that a rise in TPU hurts REC, while a less volatile trade policy supports REC. The economic interpretation of this result is that trade helps enhance REC by facilitating the trade in critical minerals, transfer of renewable energy technologies, and international collaborations. This result is in line with several past studies. For instance, Feng & Zheng [62] observed that trade is crucial for enhancing the share of REC because nations have utilized and developed renewable energy technology as they increase their bilateral trade. When capital commodities related to renewable energy are imported by industrialized countries, the use of renewable energy increases. According to Pueyo [63], trading activities also help ease the technology transfer process from advanced economies to emerging ones, which is crucial for developing renewable energy and green technologies. Wang and Zhang [48] highlighted that trade liberalization help foster REC in high-income economies. However, a negative connection exists in the context of low-income economies between trade liberalization and REC.

However, any uncertainty in trade policy or a positive shock in trading policy uncertainty restricts the flow of trade between the countries; consequently, the trade of the products used in the production of renewable energy, such as solar grids, windmills, turbines, etc., also badly impacted that have serious consequences for REC. Our findings suggest that the negative relationship between TPU and REC is more pronounced at higher quantiles, confirming that higher uncertainty attached to a trade policy lowers REC. This is not surprising because the available empirical evidence also confirms that TPU significantly impacts energy efficiency [39]. Some others have confirmed that TPU help reduce energy consumption, such as Wang and Wu [23]; Xie et al. [38]. REC is also part of total energy consumption. Therefore, the negative link between TPU and REC is justifiable.

In the long run, GDP exhibits a consistently positive relationship with REC at all quantiles. This indicates that GDP has a favorable impact on REC in China, regardless of the intensity of REC. Specifically, an escalation in GDP leads to an enhancement in REC in China. This finding aligns with Anton & Nucu [25], who documented a positive nexus between GDP and REC in the European Union. The result suggests that higher GDP tends to increase investments in renewable energy infrastructure, thus REC increases. Nyiwul [64] illustrated that high GDP per capita enables industries and individuals to afford clean and renewable energy sources, thereby increasing REC in Sub-Sahara Africa. Xu & Ullah [54] stated that environmental concerns increase due to economic progress, leading to more investments in renewable energy sources.

Regarding the CO2 estimates, only a significant positive relationship is found at quantiles 0.80th to 0.95th. This suggests that CO2 emissions contribute to REC escalation in the long run at the highest intensities of REC in China. However, no statistically significant correlation between CO2 and REC at the quantiles 0.05th to 0.70th is found. Our finding is supported by Xu & Ullah [54], who noted a positive impact of CO2 on REC in China. The study stated that as CO2 emissions increase, it raises awareness for carbon mitigating strategies, thus stimulating demand for renewable energy sources. The possible reason is that environmental pressures increase environmental regulations, thereby increasing REC.

The FD estimates exhibit a consistently positive relationship with REC from 0.20th to 0.95th quantiles. This implies that an increase in FD leads to a rise in REC in China in the long run. However, the impact of FD on REC is not statistically significant at the lowest quantiles (i.e., 0.05th and 0.10th quantiles). In the same vein, financial development has been demonstrated to be a critical factor in driving the adoption of renewable energy. Anton & Nucu [25] corroborate the positive impacts of FD on REC in the European Union. According to Ng et al. [65], financial progress may catalyze several changes, such as lowered financing expenses and monetary worries, financial capital development, and a rise in international financing, which will subsequently result in the production of goods that are more energy-conserving and state of the art technologies. Moreover, the problems of high initial costs and extended repayment periods of renewable energy businesses may be handled by applying effective financial tactics. Renewable companies may receive government assurances and bank risk incentives [66]. Therefore, the above argument supports the notion that 10.13039/501100008987FD positively impacts REC.

The short-run analysis shows that positive shocks in TPU have significant and negative impacts, at 0.20 to 0.95 quantiles. This indicates that positive shocks in TPU decrease REC in China at quantiles between 0.20 and 0.95. The insignificant effect of TPU's negative shock on REC in China has been found across all quantiles in the short term. Similarly, it has been reported that GDP estimates positively impact REC in the short term across all quantiles, indicating an increase in GDP leads to a rise in REC. Additionally, CO2 estimates positively and significantly impact REC across all quantiles in the short term, suggesting that an increase in CO2 emissions enhances REC in China. Moreover, the positive impact of FD on REC is significant from the 0.40th to 0.95th quantiles in the short term, confirming that an escalation in FD has a positive effect on REC in China.

Table 5 presents the effects of the Wald test, which was conducted to validate the existence of nonlinearities in the negative and positive components of TPU in long and short run. At all intensities in the long run, the null hypothesis of the linearity of the parameter has been rejected, which confirms the existence of nonlinearities in both components of TPU. Conversely, the null hypothesis of linearity of parameters is rejected in the short run at all intensities except 0.80th and 0.90th quantiles.

**Table 5:** Results of the Wald test.

	LR (H0: $\beta^{+}$ = $\beta^{-}$)	SR (H0: $\pi^{+}$ = $\pi^{-}$)
0.05	15.26^a^	17.23^a^
0.10	13.65^a^	15.62^a^
0.20	5.210^b^	8.512^a^
0.30	5.012^b^	8.012^a^
0.40	4.998^b^	7.998^a^
0.50	4.189^b^	6.785^b^
0.60	4.123^b^	5.658^b^
0.70	3.785^b^	2.914^c^
0.80	2.852^c^	0.432
0.90	4.125^b^	1.452
0.95	5.452^b^	3.023^c^

Note.

a p < 0.01.

b p < 0.05.

c p < 0.1.

## 3 Conclusion and implications

Trade policy is considered as a fundamental tool in the way to achieve economic interests domestically and internationally. Domestically, trade policies uncertainty can affect inflation, GDP growth, and employment. Prior studies have extensively explored the impact of trade policy uncertainties on conventional energy consumption, but none of the study have investigated the impact of trade policy uncertainties on REC. Considering this research gap, present study aims to explore the effect of trade policy uncertainty on REC in China using nonlinear QARDL. Our results suggest that an upsurge in TPU has a negative effect on REC in the short and long run. Conversely, a stable or reduced TPU only increases REC in the long run, with insignificant influence in the short run. The outcomes also show that various factors, including GDP, CO2 emissions, and financial development in the short and long term, influence REC in China. Additionally, the Wald test reveals an asymmetric effect of TPU on REC in both the short and long term.

These results may be crucial in terms of policy repercussions. Policymakers may consider the nonlinear effects of TPU on the use of renewable energy, considering both the positive and negative impacts. Given that a positive shock in TPU reduces renewable energy, while a negative shock in TPU or stability in TPU promotes REC, policymakers in China should take measures to minimize uncertainty in trade policy. Particularly, trade policies related to renewable energy sectors should be designed in a stable and predictable way. This would help in reducing the uncertainty in trade policies regarding the renewable energy sector and encourage investors to invest more money in the sector. Moreover, policymakers are encouraged to increase their international collaborations and try to sign bilateral and multilateral trade agreements with other nations and organizations. This step can be crucial in providing stability in China's trade policy and aligning the Chinese trade policies with those of other countries by controlling the risk of sudden changes that can disrupt the renewable energy markets. It is also advisable for the policymakers in China to design trade policies transparently by creating consensus among all the stakeholders, including the representatives from the renewable energy sector. This can help bring all the concerned stakeholders on board regarding government efforts in fostering REC. Further, policymakers should focus on the promotion of the domestic renewable energy industry in China, which can help reduce the reliance on imported renewable energy items. As a result, the risk associated with trade policy uncertainty will have little or no impact on the generation and consumption of the renewable energy sector. Governments, in the context of trade policies, also reduce uncertainties in financial and monetary policies as they are closely linked to trade. Financial development is also vital in improving China's demand for renewable energy. Financial institutions should provide funds for investments in the renewable energy sector.

Indeed, the study significantly contributes to literature; however, it has several limitations. For instance, the study focuses on China with regard to the nexus between TPU and REC, limiting the inferences drawn from the analysis. Although China is the major investor in renewable energy projects, but countries like the USA, Canada, and some EU economies are also investing heavily in the renewable energy sector. Thus, future researchers must focus on the role of TPU in shaping the renewable energy dynamics in some other countries as well, which would add further value to analysis. Moreover, an in-depth causal analysis between TPU and REC can prove vital in designing crucial policies. While incorporating TPU interaction terms with GDP, CO2, and financial development can enhance the comprehensiveness and depth of the study, we could not include them in our model. However, future studies can explore the potential impacts of interaction terms in analysis. In future analysis, the empirics should apply the augmented ARDL and QARDL causality test to discuss the direction association between the variables.

## 4 Data availability statement

Data will be made available on request.

## CRediT authorship contribution statement

**Qiang Zuo:** Data curation, Formal analysis, Software, Writing – original draft, Writing – review & editing. **Muhammad Tariq Majeed:** Writing – original draft, Investigation, Formal analysis, Conceptualization.

## Declaration of competing interest

The authors declare that they have no known competing financial interests or personal relationships that could have appeared to influence the work reported in this paper.
